# Combinatorial Power of cfDNA, CTCs and EVs in Oncology

**DOI:** 10.3390/diagnostics12040870

**Published:** 2022-03-31

**Authors:** Corinna Keup, Rainer Kimmig, Sabine Kasimir-Bauer

**Affiliations:** Department of Gynecology and Obstetrics, University Hospital of Essen, Hufelandstr. 55, 45122 Essen, Germany; rainer.kimmig@uk-essen.de (R.K.); sabine.kasimir-bauer@uk-essen.de (S.K.-B.)

**Keywords:** multi-parametric, multilayer, multimodal, multi-analyte, liquid biopsy, liquid profiling

## Abstract

Liquid biopsy is a promising technique for clinical management of oncological patients. The diversity of analytes circulating in the blood useable for liquid biopsy testing is enormous. Circulating tumor cells (CTCs), cell-free DNA (cfDNA) and extracellular vesicles (EVs), as well as blood cells and other soluble components in the plasma, were shown as liquid biopsy analytes. A few studies directly comparing two liquid biopsy analytes showed a benefit of one analyte over the other, while most authors concluded the benefit of the additional analyte. Only three years ago, the first studies to examine the value of a characterization of more than two liquid biopsy analytes from the same sample were conducted. We attempt to reflect on the recent development of multimodal liquid biopsy testing in this review. Although the analytes and clinical purposes of the published multimodal studies differed significantly, the additive value of the analytes was concluded in almost all projects. Thus, the blood components, as liquid biopsy reservoirs, are complementary rather than competitive, and orthogonal data sets were even shown to harbor synergistic effects. The unmistakable potential of multimodal liquid biopsy testing, however, is dampened by its clinical utility, which is yet to be proven, the lack of methodical standardization and insufficiently mature reimbursement, logistics and data handling.

## 1. Introduction

To date, clinical diagnosis of malignant disease is still largely reliant on imaging technologies, such as X-ray, computer tomography, magnetic resonance imaging and ultrasound, requiring a certain minimal diameter for suspicious lesions to be detected. However, disease stage at the time of diagnosis is a strong indicator of patient prognosis [1]. It seems plausible that the detection of malignant disease at an earlier stage would improve patient prognosis by enabling personalized treatment regimens based on dedicated prognosis and molecular characterization of the tumor.

The potential of the analysis of body fluids, called liquid biopsy, has recently become omnipresent in translational oncology. In different tumor entities, liquid biopsy analysis has proven to enable highly informed decisions in the different challenging situations of cancer management. Early cancer, minimal residual disease and metastasis detection by liquid biopsy analysis is possible before the tumor mass is detectable via imaging techniques, because the tumor cell characteristics and changes in the systemic balance can sensitively be detected [2,3]. Regarding the personalized treatment, collection of body fluids has the advantage of being non-invasive or minimally invasive, and thus, liquid biopsy profiling is repeatable and mirrors the temporal changes under therapy—defined as tumor evolution [4]. In addition, liquid biopsies can mirror the entire tumor heterogeneity that occurs in systemic oncological situations. In the case of tumor masses that are difficult to biopsy, liquid biopsy is the only option to molecularly characterize the tumor in these locations [5].

In summary, liquid biopsy analysis has a vast potential to revolutionize medical oncology.

## 2. How Diverse!—The Repertoire of Liquid Biopsy Analytes

A variety of different body fluids are usable for liquid biopsy analysis. Urine and saliva can be obtained non-invasively, and urine was shown to be suitable for the diagnosis of prostate cancer (PC) [6] and for mutation detection in bladder cancer [7]. Saliva testing is useful for virus and mutation detection in head and neck cancer [8]. Blood can be obtained minimally invasive and is the liquid biopsy reservoir most commonly studied in the last years. As all tumor masses are highly vascularized, blood mirrors the systemic tumor disease in almost all tumor entities [9]. An exception are brain tumors—in these, cerebrospinal fluid can be used as body fluid to monitor the genomic tumor evolution [10]. Bile was recently shown to provide mutation information in biliary tract cancer [11]. Proteins in breast milk were used to detect breast cancer (BC) [12]. Genomic information from pleural effusions in lung cancer patients was more accurate compared to plasma information [13]. Tumor cells can be obtained and characterized from ascites in ovarian cancer patients [14] and patients with gastrointestinal cancers [15]. Highly innovative methods have been developed to potentially detect cancers based on extracellular vesicles in tears [16], to detect lung cancer based on bronchial lavage fluid [17] and to detect PC using cell-free DNA concentrations in seminal fluid [18].

**Figure 1:**
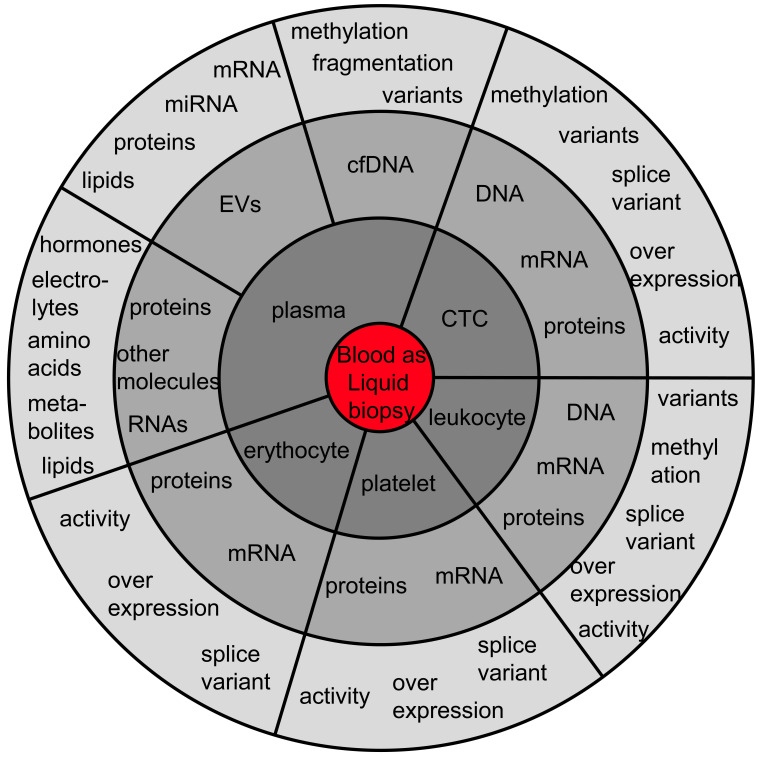
**The diversity of liquid biopsy analytes in blood.** The analytes can be separated into tumor cells, non-malignant blood cells and analytes within the plasma fraction. Not all of the mentioned analytes are tumor-derived/specific, but all analytes can provide information about the tumor-associated systemic changes mirrored in the blood stream. The authors do not guarantee completeness of the list of blood analytes usable as liquid biopsy reservoirs in oncology. Abbreviations: cfDNA: cell-free DNA; CTC: circulating tumor cell; EVs: extracellular vesicles.

Regarding blood, it is important to recognize that dozens of analytes are usable for cancer management (Figure 1). Here, the different liquid biopsy analytes are listed, with their potential clinical feasibility highlighted.

### 2.1. Circulating Tumor Cells

Tumor cells that migrate into the blood stream are called circulating tumor cells (CTCs). They can adapt to the conditions via epithelial-to-mesenchymal transition (EMT) to finally invade again and form metastases. As CTCs are part of the metastatic cascade, their characteristics are of relevance for therapy intervention. Enumeration of epithelial CTCs was shown to be of prognostic value in solid tumors [19]. The epitopes on CTCs provide molecular information that is potentially relevant for targeted therapies. Consequently, among others, the HER2 protein [20], the PD-L1 protein [21,22] and the androgen receptor (AR) protein [23] were examined on CTCs. CTC mRNA was frequently studied with either a focus on the overexpression of specific transcripts [24,25,26,27] or the presence of specific splice variants [28,29,30]. The latter was shown to have therapeutic relevance in PC patients, as the AR splice variant 7 was detected in CTCs and directly correlated with resistance to anti-AR treatment [31]. Lately, the isolation and characterization of variants and methylation of CTC genomic DNA became possible. Single-nucleotide polymorphisms (SNPs) in CTCs were studied via allele-specific PCR [32], crystal PCR [33], Sanger sequencing [34], targeted sequencing [35] and many other methods. Copy number variations (CNVs) were examined in CTCs via whole-genome sequencing [36,37]. Moreover, DNA methylation of CTC gDNA has already been studied [38,39]. More advanced techniques were required to even identify information in single CTCs—not pools of CTCs. Sensitive enrichment and specific identification of single CTCs is challenging, but emerging techniques from material science include, i.e., magnetic silica nanoparticles coupled to antibodies and fluorescent probes [40], antibody-mediated magnetic surface-enhanced Raman spectroscopy [41] and nanotags for other specific microscopy techniques conjugated with antibodies [22] to solve the sensitivity and autofluorescence problems. Single CTCs were successfully sequenced [42,43,44], their epigenomic information was obtained [45], transcriptional signatures identified [46] and even their function was examined [47]. With the blood of patients harboring a huge CTC count, it became possible to establish CTC cell lines [48] and xenografts [49], which are potentially relevant to test drug sensitivity for individual patients.

### 2.2. Cell-Free DNA

DNA is released into the blood stream by non-malignant and malignant cells, mostly through apoptosis. The rapid turn-over of malignant cells and potentially also apoptosis of therapy-sensitive tumor cells contribute to the fraction of tumor-originating DNA in the entirety of cell-free DNA (cfDNA) [50]. The tumor-originating DNA is generally characterized by the presence of variants and is called circulating tumor DNA (ctDNA). The genomic alterations in cfDNA revealed not only prognostic value [3], but revealed genomic evolution under therapy [51]. Mutational testing of cfDNA has already been translated into clinical practice for therapy guidance, i.e., *EGFR* testing in lung cancer [52] and *PIK3CA* testing in BC [53]. Many more clinical studies showed the benefit of cfDNA variant testing for personalized treatment [54,55]. Sensitive methylation analysis of 5-methylcytosines in cfDNA was shown to enable early cancer detection [56] and minimal residual disease detection [57,58]. The methylation analysis of 5-hydroxymethylcytosine could be used to identify the tissue of origin and differentiate tumor stages [59]. Besides variant and methylation information of cfDNA, the length of cfDNA fragments and their end motifs [60,61] can provide an additional dimension of information. The fragmentation length was shown to differ in wild-type DNA fragments compared to mutant fragments [62,63]. Fragmentation and sequencing coverage at transcription start sites might give a hint as to the nucleosome accessibility in the cells of origin. Thus, this DNA analysis approach could be used to identify the expression status of specific genes and the tissue of origin of the ctDNA fragments [64].

### 2.3. Extracellular Vesicles

Extracellular vesicles (EVs) enable intercellular communication [65]. These lipid-bilayer-enclosed compartments, ranging in size, can be released by malignant and non-malignant cells [66,67]. The entirety of EVs in the blood provides insights into the systemic oncologic setting, including the changes in tumor-associated processes mediated by the immune system [68]. EVs contain molecules from the cell of origin, mostly RNAs and proteins [69]. Besides protein-coding RNAs, tRNAs, microRNAs, large intergenic ncRNAs and small nucleolar RNAs (snoRNAs) were sequenced in plasma [70]. EV mRNA is protected from degradation within the vesicles and has previously been described as useful for prognosis [71] and therapy monitoring [27] in solid tumors. MicroRNAs (miRNA) are also enclosed in EVs, and it has been shown that EV miRNA profiling in urine can be used to screen for PC [6]. Integrins on the EVs surface condition the pre-metastatic niche [72] and could thus, provide information on the potential location of metastases. The protein content on the vesicle surface partly mirrors the surface markers of the cell of origin. In BC patients, HER2 proteins were found on EVs [73]. This observation can, on the one hand, provide an argument for anti-HER2 treatment in patients with HER2-negative primary tumors, but on the other hand, can also explain why the anti-HER2 treatment is not effective: anti-HER2 antibodies bind to the HER2-positive EVs rather than inhibiting the HER2 signal activation on the tumor cell surface [74]. Specific multiplexed assays allowed the detection of multiple exosomal surface epitopes and enabled the differentiation of PC patients compared to healthy controls [75]. Sensitive FACS analysis can also be used to enumerate EVs expressing specific surface markers in a multiplexed matter [76]. Whether the lipid composition of EVs can also be used for clinical management of tumor patients is to be discussed [77]. Independent of the usability of EVs in the field of liquid biopsy, engineered EVs can be loaded with specific siRNAs or toxic components and can be modified with surface markers to be incorporated only by tumor cells, thus acting as specific vehicles to deliver therapies [78].

### 2.4. Circulating RNAs

RNAs circulating in the blood stream and not protected by the lipid bilayer of an extracellular vesicle are prone to degradation. However, the association of RNAs with proteins (such as the Argonaute2 complexes or high-density lipoproteins) impede the degradation as well, and thus, circulating RNAs can be found [79,80]. The different species of RNA in blood, as well as in other body fluids, were recently studied in detail in samples from different diseases [81], showing that circular RNAs were increased in most body fluids. MiRNAs, as part of the epigenome, can drive tumor genesis by regulation of chromatin structure and gene expression [82]. Circulating miRNAs can be detected in the blood without enrichment for EVs. Specific miRNA signatures were shown to differentiate BC patients from healthy donors [83,84] but can also be useful as prognostic markers for neo-adjuvant treatment in BC patients [85]. Another dimension of the miRNA biology, the methylation of miRNAs, has been shown to enable early detection of pancreatic cancer via blood analysis [86].

### 2.5. Circulating Proteins

Blood-based protein markers have been well-established in clinical practice to quantify tumor responses. In this regard, serum PSA levels were used to monitor the response to treatment of bone metastases in PC [87]. Carcinoembryonic antigen (CEA) and carbohydrate antigens (CA) 125, CA 29, CA27, CA 19-9, CA 15-5 and CA 15-3 were discussed for therapy monitoring in BC patients, as well as in direct comparison with the monitoring value of other blood analytes [88,89]. The long half-lives of the circulating proteins might be one reason why circulating proteins mirrored the therapy response less sensitively compared to, i.e., *PIK3CA* mutations in cfDNA of BC patients [90] and the tumor fraction in the cfDNA [89].

Sensitive quantification of low-abundance proteins in the circulation is a technical challenge that was recently addressed with nanoparticle-based enrichment and subsequent mass spectrometry [91,92,93,94]. Moreover, enrichment of metalloproteins with magnetic chelating nanoparticles [95] or enrichment and detection of serum proteins with fluorescent nanocrystal microbeads coupled to a variety of antibodies [96] was described. An even more advanced technique with easy clinical application is the instrument-free, cost-effective method to enrich and colorimetrically detect phosphopeptides from serum samples within 15 min using TiO_2_-nanoparticle-based chemisorption and a tetrabromophenol assay [97].

BC diagnosis was determined to be achievable with afamin, apolipoprotein E, alpha-2-macroglobulin and ceruloplasmin [98] or the integrin subunit alpha, Filamin A, Ras-associated protein-1A and Talin-1 [92]. Ovarian cancer diagnosis was shown to be promising with just a single circulating protein, the hepatocyte growth factor [99]. However, how tumor-specific the circulating protein analysis is remains unknown. These considerations evolved from the knowledge of PC detection based on circulating PSA testing, which resulted in massive over-diagnosis and over-treatment due to false-positive results [6,100]. However, the usage of models predicted by artificial intelligence including multiple conventional proteins could overcome the low specificity [101]. Recently, the activity of circulating thymidine kinase 1 (sTK1) acquired new importance as a cell cycle marker to examine the efficacy of oncological treatment [102]. Similarly, the circulating immune checkpoint proteins PD-1, PD-L1 and CTLA-4 recently gained attention [103,104]. Regarding the epigenomic dimension of post-translational protein modifications, it might be worth further exploring the value of histone modifications and associated cfDNA, as previously conducted with chromatin immunoprecipitation of cell-free nucleosomes followed by sequencing [105,106].

In summary, circulating proteins have to be discussed with caution as they are potentially no specific tumor markers and thus, are not included in the classical field of liquid biopsy. The same holds true for clinical chemistry blood analytes, such as hormones, amino acids, metabolites, electrolytes and lipids—these molecules are not classically integrated in the list of liquid biopsy analytes, despite their advantage of possibly quick implementation in clinical practice [101], which is in contrast to the highly sophisticated genomic analyses of cfDNA or CTCs.

### 2.6. Blood Cells

With regard to the emerging immune therapies and the rising awareness about the tumor microenvironment and peripheral immunological processes, it is obvious that blood analytes, not directly derived by malignant cells, have gained importance recently [107]. The ratios of the different blood cells (neutrophil-to-lymphocyte, NLR; monocyte-to-lymphocyte, MLR; platelet-to-lymphocyte, PLR) might be used to help profile the immunological situation and corresponding prognosis and treatment outcome [108,109]. A preprint described an altered proteomic composition of red blood cells of metastatic BC (MBC) patients compared to healthy donors. The LAMP2 protein in the red blood cells significantly correlated with tumor stage and prognosis [110], thus, highlighting alterations in non-malignant components during a systemic oncological disease. It is to be speculated whether the DNA-sensing protein TLR9 on the surface of red blood cells [111] is triggered by a massive increase in cfDNA during tumor disease and whether this might lead to altered proteomic composition in mature red blood cells. Variants in splice sites were found to cause accumulation of specific elongated mRNA transcripts in red blood cells and led to inherited RBC disorders [112]; thus, it should be discussed whether some splice variants in red blood cells might serve as markers for cancer management.

The cell–cell interaction of tumor cells and platelets was shown to alter the signaling cascades of platelets, then called tumor-educated platelets [113]. Consequently, the analysis of platelet RNA was proposed for multicancer detection [114] and therapy monitoring [115]. It is still unknown whether specific splice variants can be transferred from tumor cells to platelets and might specifically be detected due to the very low prevalence of splice variants in platelets of healthy donors [116].

Finally, on the one hand, leukocytes can serve as germline control, in contrast to the somatic variants in tumor cells, but on the other hand, specific germline polymorphisms were also associated with therapy sensitivity [117], involvement of the immune system [118] and susceptibility to adverse effects from chemotherapies [119]. In addition, leukocyte DNA methylation might be interesting to study, due to the relevance of epigenomic dynamics, especially in cells highly adaptive to the environment [120]. Recently, blood cell DNA methylation patterns were used to normalize for non-malignant methylation patterns [121]. Another fascinating field is the T-cell receptor (TCR) repertoire, which is the number of T-cells with different T-cell receptors, screened via RNA-seq of the variable region CD3 [122]. It has previously been shown that a large TCR repertoire leads to an increased benefit of immune checkpoint inhibitors (ICIs) [123]. Since the TCR repertoire in the blood was shown not to represent the TCR repertoire in the tissue [124], the value of TCR sequencing via blood samples still has to be examined [107]. The number of circulating immune cells with specific surface proteins can also give us a hint regarding therapy response; i.e., increased numbers of circulating CD4+ CD25+ CD127– FOXP3+ cells were found in BC patients experiencing tumor progression [125].

## 3. Revealing the Best One?—One Liquid Biopsy Analyte versus Another Liquid Biopsy Analyte

The diversity of blood liquid biopsy analytes allows for the selection of the most suitable one for a specific clinical purpose from a broad repertoire of analytes. Therefore, analytes were directly compared in matched samples to find the most eligible marker (Table 1).

### 3.1. CTC Enumeration and cfDNA Levels

Basic techniques in the liquid biopsy field include the enumeration of CTCs and the concentration determination of cell-free DNA. These two analyses were frequently conducted for direct comparison in BC patients: compared to CA15-3 and the number of CTCs, cfDNA analyses showed a broader dynamic range and a more sensitive correlation with disease burden [88]. The total cfDNA level, but not the CTC count, was a predictor for PFS and for the progression time point [135] in MBC patients. Moreover, it was shown that the dynamics in ctDNA levels after two cycles of paclitaxel and bevacizumab were more strongly correlated with overall survival (OS) compared to the CTC count in HER2-negative MBC patients [136]. In contrast to the two studies mentioned above, Shaw et al. and Fernandez-Garcia et al. concluded that CTC count and total cfDNA level were both equally valuable markers for OS in MBC patients [128,135]. Many studies concluded that the combination of CTCs and cfDNA is more informative than a single analyte for prediction of OS [135,137] and PFS [137].

### 3.2. Matched CTC and cfDNA Variant Profiling

The molecular characterization of blood analytes is more advanced (Table 1) than CTC enumeration and cfDNA level determination. This is particularly relevant because CTCs can invade secondary organs as seeds of metastasis [138] and are therefore, active components in the metastatic cascade. In contrast, cfDNA is released by passive processes such as apoptosis and necrosis, mostly by non-malignant cells [139] and therefore, at the moment, cfDNA is regarded as a liquid biopsy marker, rather than a functional component in the oncological setting. CTC isolation is still not standardized, and both CTC isolation and mutation analysis are, in general, challenging with regard to sensitivity and specificity. In contrast, cfDNA does not have to be enriched for the tumor-derived fraction [140], can easily be quantified and sensitive variant calling was developed for ctDNA [141]. One example for the latter statement is the direct comparison of CTC and cfDNA mutations in BC patients [29]. Beije et al. showed a higher sensitivity of *ESR1* mutation detection in cfDNA compared to CTCs. At the progression time point under endocrine treatment, the difference between the mutation detection rate in cfDNA compared to CTCs was the highest (42% patients with *ESR1* mutations in cfDNA; 11% patients with *ESR1* mutations in CTCs). In a small cohort of five MBC patients, the cfDNA mutations reflected the mutations detected in their single CTCs. However, in two patients, more mutations were detected in cfDNA than in the single CTCs [128]. We can conclude that a large number of single CTCs has to be analyzed to identify the range of mutations present in a systemic oncological situation such as MBC. However, only CTC mutation profiling and not cfDNA mutation profiling can reveal newly emerging resistance mutations under treatment [129]. Additionally, only CTCs depicted the subclonal evolution in a longitudinal case study of an MBC patient over four years [126] and in five HER2-mutant MBC patients [127].

**Figure 2:**
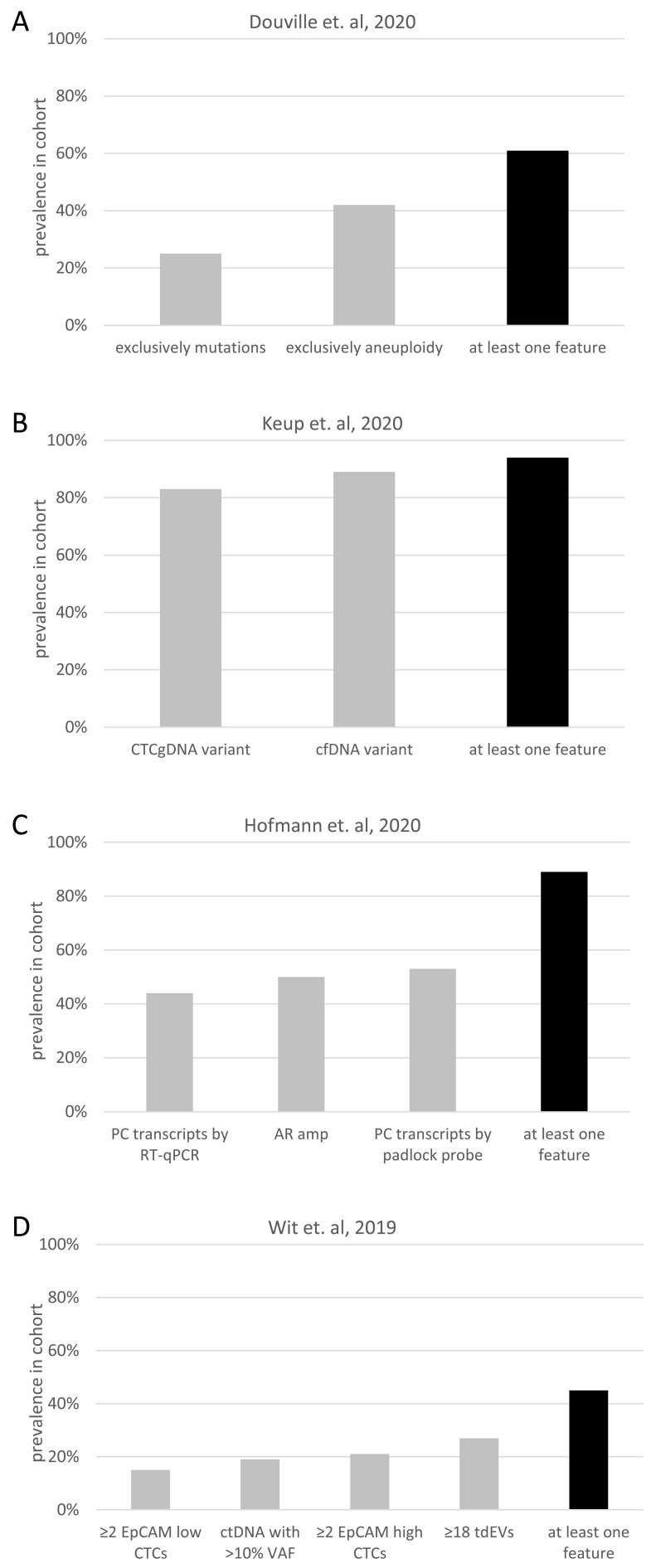
**Additive value of multiple liquid biopsy analytes.** The prevalence of different molecular features and the prevalence of at least one of the different features is depicted, based on data of four publications: (**A**) [143], (**B**) [35], (**C**) [144] and (**D**) [145]. Abbreviations: AR: androgen receptor; Amp: amplification; cfDNA: cell-free DNA; CTC: circulating tumor cell; ctDNA: cell-free tumor DNA; EVs: extracellular vesicles; gDNA: genomic DNA; PC: prostate cancer; RT-qPCR: real-time quantitative PCR; tdEVs: tumor-derived EVs; VAF: variant allele frequency.

The concurrence of CTC and cfDNA mutations was shown to depend on the tumor stage, with a lack of concurrence in early BC patients, but with increased concurrence in MBC patients [130]. Thus, the complementary nature of CTCs and cfDNA was described in early BC patients [130]. Within the same year, a method for cfDNA mutation profiling from CTC-depleted blood [142] to molecularly characterize cfDNA and CTCs from only 10 mL of whole blood from MBC patients was developed. Direct comparison of mutations in cfDNA and CTCs revealed the complementary nature of these analytes in the metastatic situation, due to a small overlap (28%) of identical mutations in both fractions [35]. The majority of cfDNA variants were also recovered in the matched CTC gDNA, while 72% of all variants were unique in either cfDNA or CTC gDNA. Only 6% of the patients showed neither cfDNA nor CTC variants, while the percentage of patients without any detectable cfDNA variants or CTC gDNA variants was 17% and 11%, respectively (Figure 2B). Interestingly, *ERBB2* variants were only detected in CTC gDNA. *BRCA1* and *BRCA2* variants were more frequently found in CTCgDNA than in cfDNA. Additionally, *PIK3CA* and *ESR1* mutations were more common in cfDNA compared to CTCgDNA. Variants in all of these five genes might indicate targeted therapy options; thus, the comprehensive mutational analysis of cfDNA and CTCs can maximize the number of patients in whom actionable targets can be identified [35].

### 3.3. Matched CTC and cfDNA Methylation Profiling

In addition to variant profiling, DNA methylation profiling has already been conducted in matched cfDNA and CTCs. The *SOX17* promotor methylation in cfDNA and CTCs significantly correlated with each other in early BC [131] and MBC patients [132]. Moreover, *SOX17* promotor methylation in cfDNA and CTCs was shown to significantly correlate with OS in MBC patients [132]. *ESR1* methylation in cfDNA and CTCs was concurrent in 98.3% of BC patients (n = 58) [39]. Additionally, only the *ESR1* methylation in CTCs, not in cfDNA, was associated with a lack of response to an everolimus–exemestane regimen [39].

It is important to note that the combination of 5-methylcytosine (5 mC) and 5-hydroxymethylcytosine (5 hmC) analysis in cfDNA significantly improved the sensitivity of pancreatic cancer detection [133] compared to the analysis of only one methylation type.

### 3.4. Matched CTC and EV Characterization

Direct comparison on protein, the RNA and the DNA level can be conducted with matched CTCs and EVs. Based on protein staining, both the number of HER2-positive (HER2+) and Cytokeratin-negative (CK-)- CTCs and HER2+ CK- EVs were found to correlate with the time of blood draw to death in MBC patients [73].

In PC patients, the presence of the androgen receptor (AR) variant seven, an important marker against choosing anti-AR therapy [31], was more common in CTCs as compared to matched EVs [134].

In 2018, matched CTCs and EVs from the same blood sample were molecularly characterized for the first time [27]. Only 5% of the signals were identical in both fractions, representing a great difference between CTC and EV mRNA profiles in the matched samples from MBC patients, demonstrating the complementary nature of the analytes. Interestingly, the overexpression of the *mTOR* transcript in CTCs was associated with response to therapy, while the *mTOR* transcript signals in EVs were more frequently present in non-responders. Thus, the same transcript in either CTCs or EVs was associated with contrary clinical outcomes. Another observation was the synergistic value of CTCs and EVs regarding the signals of the ERBB protein family, which is explained by the stronger correlation of *ERBB2* and *ERBB3* signals in CTCs and EVs with disease progression compared to *ERBB2* and *ERBB3* signals in CTCs alone [27]. These results confirm the hypothesis that the information depicted by a single blood analyte is limited.

## 4. Additive Value!—The Combination of Three or More Liquid Biopsy Analytes

Most of the presented studies, comparing two matched liquid biopsy analytes, showed a similar value of the analytes or a benefit of a combined analysis. However, the comparisons were only conducted on either the DNA, RNA or protein level; a multi-omic approach, integrating information on different levels of evidence, might be even more informative. In addition, due to the great diversity of analytes, it should be investigated whether a combination of more than two analytes might even more comprehensively reflect the molecular picture of the disease. To verify this hypothesis, some groups have already addressed this issue by conducting multimodal liquid biopsy studies on different tumor entities with different clinical purposes, which are detailed in the following (Table 2).

### 4.1. Technical Feasibility

The query about the technical feasibility of a multimodal analysis of CTC and leukocyte enumeration and cfDNA variant analysis in healthy donors revealed methodological challenges. In this context, the blood collection tube, blood storage time and sample preparation methods were shown to influence the results [146]. The importance of the choice of the blood collection tube was also the quintessence of a multimodal liquid biopsy study, including CTC enumeration, cfDNA variant analysis and vesicular as well as non-vesicular miRNA profiling, in melanoma patients [147].

### 4.2. Early Cancer Detection

Aiming for early cancer detection, multimodal liquid biopsy testing exhibits huge potential due to the improved sensitivity by combining orthogonal data sets [50]. This hypothesis was proven by mutation and aneuploidy testing in cfDNA (Figure 2A) combined with profiling of eight circulating proteins to detect solid cancers with higher sensitivity (80% at 99% specificity) compared to the separate analysis of the analytes [143]. Early cancer detection via multimodal liquid biopsy testing also became more sensitive (91% at 98% specificity) when combining cfDNA SNV, CNV and fragmentation analysis [148]. In a smaller cohort (n = 205), information for cancer detection was also shown to be additive from the distinct data sets of selected cfDNA mutations, promotor methylation patterns and miRNA expression level [149]. However, the fragile balance of sensitivity and specificity was described in two of the three mentioned multimodal liquid biopsy cancer detection studies. They concluded that an ideal number of analytes exists, while adding more analytes would increase sensitivity, but reduce specificity [2,149]. Commercialization of multimodal liquid biopsy early cancer detection tests is becoming popular, and several large companies announced collaborations to realize this ambitious goal.

Besides the early cancer detection of several solid cancers and detecting the tissue-of-origin from a single blood sample, multimodal liquid biopsy analysis might help in testing for specific tumor diseases. Methylation and CNV profiling in cfDNA and quantification of the protein CEA in the same blood sample identified twice as many colorectal cancer patients as single analyses—adding up to a 94% sensitivity and specificity [150]. Moreover, the usage of cfDNA fragment size selection combined with CNV and fusion gene analysis was shown to be useful for early cancer detection in entities not driven by SNVs—such as childhood cancers in general and Ewing sarcomas in particular [151], because fragment-size filtering of cfDNA profiles enhanced the CNV detection.

### 4.3. Prognostification

In a proof-of-concept study, a multimodal liquid biopsy approach was evaluated for initial staging of pancreatic cancer patients [152]. The training set of 47 subjects revealed a combination of 14 candidates, including miRNA/mRNA, KRAS mutation, cfDNA concentration and CA19-9, isolated from 3 mL of whole blood as a promising panel. This combination then achieved a sensitivity of 88% and a specificity of 95% in the validation cohort [152].

In the context of pancreatic cancer, another group evaluated the usage of cfDNA concentration, RAS mutations in cfDNA and the ratios of neutrophils or platelets to lymphocytes for prognosis prediction [153]. They showed NLR to correlate with PLR, and found a correlation between NLR, cfDNA levels and the RAS mutation level. Combining NLR, PLR, cfDNA levels, RAS mutation presence, RAS allele fraction and CA19-9 increased the certainty of the prognostic statement in metastatic pancreatic cancer patients [153]. Therefore, the use of systemic inflammatory markers together with circulating tumor-specific markers presents a valuable tool for clinical management.

In nearly 100 lung cancer patients, CTCs, tumor-derived EVs and ctDNA were examined [145]. While all these biomarkers were present in only 2% of the patients, at least one of these analytes was detected in 45% of the patients (Figure 2D). Moreover, the presence of two or more of these biomarkers was associated with poor OS [145].

In 62 PC patients, CTC mRNA and EV mRNA profiling revealed a greater number of tumor-associated mRNA profiles of eight genes in CTCs compared to EVs [154]. While *GSTP1* methylation in CTCs and EVs was significantly associated with OS, *RASSF1A* methylation was only prognostic in EVs, and *CK19*, *PSMA* and *TWIST1* mRNA expression were prognostic markers only in CTCs [154].

In the so-called ELIMA project, the value of cfDNA, CTC gDNA, CTC mRNA and EV mRNA for prognostication and therapy guidance was elucidated in MBC patients [155]. This multi-omic approach aimed to simultaneously analyze the transcriptional and genomic complexity from only 20 mL of blood. Regarding prognostication, the combination of clustering results of the single analytes to the ‘ELIMA.score’ resulted in a significant correlation with a decreased p-value for OS when compared with the clustering within each single analyte. Importantly, some information within the four analytes overlapped, but all of the analytes added information to the global multimodal data set that was absent in the other analytes. Consequently, no analyte alone was sufficient to comprehensively reflect each individual disease, but CTC gDNA, CTC mRNA, EV mRNA and ctDNA were complementary.

### 4.4. Therapy Guidance

Furthermore, multimodal liquid biopsy approaches might be used for early disease recurrence identification and consequently, potential escalation of the adjuvant therapy. In contrast to the single approaches, the combination of CTC enumeration and cfDNA mutational profiling resulted in the highest sensitivity rates in predicting minimal residual disease in triple-negative BC patients [156].

For both early and metastatic BC patients, it was shown that the comprehensive evaluation of CTCs via their mutation, methylation and mRNA expression levels has advantages over CTC identification only based on protein staining [157]. Thus, combinational CTC testing was discussed as a powerful strategy for molecular characterization of the individual disease.

The potential of a multimodal approach as to ensure that ‘no information is left behind’ was included in the marketing approach of Epic Science. They announced on 16 December 2021 that they would use CTC presence, their ER and HER2 protein status and *ERBB2* amplification presence, as well as the CNV, SNV, fusion and microsatellite instability analysis and tumor mutational burden quantification in cfDNA, called ‘DefineMBC’, to classify the different BC subtypes and enable therapy guidance.

Detailed molecular characterization of individual MBC patients via multimodal liquid biopsy testing was also conducted in the ELIMA project. Here, the final integration of the information from matched CTC mRNA, EV mRNA, cfDNA and CTC gDNA, assessed at disease progression in MBC patients, resulted in an increased number of patients with actionable signals, which is useful for personalized therapy decisions in comparison with the assessment of only a single analyte [155].

Concerning therapy decision management in PC patients, it was shown that the expression of variant seven of the AR transcript resulted in resistance to AR inhibitors [31]. Combinational analysis of transcripts (*AR full length*, *AR variant 7* and *PSA*) in CTCs revealed that the same transcripts in whole blood and focal amplifications of AR in cfDNA led to the identification of resistance mechanisms developed prior to therapy in a larger fraction of patients when compared to the evaluation using a single analyte [144] (Figure 2C). Another group collected the data of multiple liquid biopsy analytes (CTC count, CTC CNVs and SNVs, cfDNA variants and cfRNA) from two PC patients. They concluded that the distinct data sets validate the analytes’ orthogonal character, which is potentially explained by true biological distinctions, but also highly influenced by methodological factors [158].

The multi-analyte approach, called DIREct-On, in lung cancer patients will be suitable for clinical routines in the future, as it is a predictive panel supporting therapy decisions for ICI therapy in these patients [159]. The approach integrates the number of peripheral T-cells, blood-based tumor mutational burden and levels of ctDNA. Each analyte is required for optimal differentiation between responders and non-responders and the predictive marker combination obviates the need for PD-L1 status evaluation in the tissue for therapy guidance.

### 4.5. Therapy Monitoring

In the latter study, the levels of ctDNA were not only determined before treatment initiation, but also after a single cycle of therapy [159]. The authors concluded that adding an on-treatment measurement of cfDNA improved the prediction for those who will benefit from the given ICI therapy. The purpose of therapy monitoring (currently via imaging techniques, but in the future possibly by liquid biopsy), is to deescalate, escalate or change the treatment regimens to achieve the best possible patient outcome.

Longitudinal blood draws (before and under a new therapy regimen) were conducted in MBC patients [160]. The multimodal approach with CTC mRNA, EV mRNA and cfDNA underscored the additional value of these analytes under treatment and stated unique features of the single analytes for disease monitoring [160].

The advantage of single CTC analysis in a longitudinal manner was presented by an MBC case study with 17 blood samples [126]. In general, CTC count, cfDNA concentration and the tumor fraction within the cfDNA entirety correlated with each other and were increased at disease progression. The authors hypothesized that the tumor might have developed due to CNVs (found in tissue, CTCs and cfDNA), while disease progression might have developed due to SNVs (detectable in cfDNA and CTCs but not in the tissue). However, in cellular resolution, the tumor evolution under treatment can only be mirrored by the analysis of single CTCs [126,127].

## 5. What Now?—Challenges to Be Solved in the Future

Multi-analyte liquid biopsy testing is promising for clinical practice in the future. However, the multi-analyte studies were conducted in the translational space and until now, no prospectively randomized trial has included multi-analyte liquid biopsy. Only with international multicenter interventional trials in large cohorts, with the intervention based on multi-analyte testing (in comparison to single analyte testing), can clinical utility be proven.

The methodical challenges mentioned below are important for the design of clinical trials, as well as considerations about the biological and life style factors of each patient, which may influence the liquid biopsy results. In this regard, it has already been shown that physical exercise influences the levels of EVs [161] and cfDNA [162,163], while the circadian rhythm was purposed to influence the number of CTCs and other circulating cells [164]. Consequently, controlling for physical activity and time of the day before/at blood draw should be included in the study design, with the consideration of many more factors, such as sex, age, diet, obesity, hypertension, stress, smoking and alcohol consumption. In general, the lack of understanding of the biological mechanisms of release and degradation of the analytes impedes the analytical robustness [163].

Another huge challenge in the multimodal liquid biopsy field is the lack of methodical standardization. In this context, preanalytical processes, such as blood collection tubes and blood storage, were shown to have a great effect on the final results [146,147]. Furthermore, the analytical processes are highly diverse. Different analysis methods were used for protein, RNA and DNA, and different isolation methods were used for EVs, CTCs and cfDNA. Various PCR and sequencing techniques and, in general, a miscellaneous collection of analytes within the multimodal approaches, exist. Consequently, direct comparison of multimodal data sets is not possible, and reproducibility is lacking. However, the final conclusions of these studies resemble the combinational power of the analytes. It is almost not conceivable that the analytical processes in different multimodal studies can be standardized. However, endeavors at least should be undertaken to standardize single analyte workflows (such as cfDNA isolation and sequencing [165]). Along with the need for standardization is the need for automation. It would reduce the variability caused by different experimenters, and human mistakes would be eradicated.

In addition, the blood input amount needed for sensitive multimodal testing should be as low as possible to increase the patient compliance. The field can only move forward with an innovative decentralized infrastructure for sample collection and effective and quick shipping to the central laboratory or even with decentralized testing to enable preanalytical variation and turn-around time to be minimized.

Furthermore, especially because of the large number of data produced in each multimodal analysis, the post-analytical processes should be reconsidered. Not only should data handling in terms of privacy and storage be rethought, but data interpretation and readability for the users as well. In this regard, the data integration and interpretation by artificial intelligence should especially be examined, as deep learning approaches might save time, prevent human mistakes and might outperform classical-software-based approaches that are on the market, at the moment. At the interface of data interpretation and readability for the clinicians, it has already been shown that different tools show different treatment recommendations based on the same blood results [166]. Consequently, it will be important to update the level of evidence of specific test results [167] based on the current findings in clinical trials and standardize the evidence levels worldwide and beyond tumor entities. Finally, integration of these updated levels of evidence in all available tools for data interpretation is needed. In the last step, clarity of the treatment decisions recommended based on the blood results should be ensured by user-friendly design of the final output.

Obviously, the combination of multiple analyses increases the costs of the multimodal testing. Reimbursement of these costs has to be assured before multimodal testing can be offered to all patients. As mentioned above, the additive value of the combinational approaches is mainly the increased sensitivity; thus, single sequential tests for different potential diagnoses are unnecessary and earlier cancer detection is enabled. These issues point towards the advantage of multimodal testing despite the increased costs. The cost-effectiveness for companion diagnostics in general has already been shown [168].

The advantages of multi-analyte liquid biopsy testing can only be transferred into clinical practice in the case that these challenges can be overcome [169]. Until then, multimodal liquid biopsy studies in the translational field will combine the most different markers in different clinical scenarios and might either find the most suitable analyte for a specific clinical purpose or might validate the combinational power of cfDNA, CTCs and EVs in oncology.

## 6. Conclusions

The diversity of liquid biopsy analytes in the blood for clinical management of cancer patients is incredible. Direct comparison of two liquid biopsy analytes showed some analytes to be more suitable for a specific clinical purpose than others. Within the last three years, multi-analyte liquid biopsy studies have been introduced, and more than a dozen of these projects have been published to date. In every multimodal liquid biopsy study, cfDNA was evaluated, but the additional components were as diverse in the different studies as the clinical purposes and tumor entities. A particularly promising approach that is not too far from realization in clinical routines is early cancer detection via the molecular characterization of different blood components, as the sensitivity of this approach dramatically increased with the usage of more than one analyte. Although the additive value of multiple analytes for prognostication and therapy monitoring was shown, the authors believe that—as a start—multimodal liquid biopsy should be considered as the first priority for therapy guidance in the era of personalized medicine. Translational studies have already demonstrated that targeted therapy decisions can be enabled for a higher proportion of patients in the case diverse blood analytes are characterized. Large prospective trials with interventions based on multimodal liquid biopsy should be carried out to solve the biggest challenge for implementation: the still unknown clinical utility.

## Figures and Tables

**Table 1:** **Molecular characterization of two liquid biopsy analytes from studies describing their direct comparison in matched samples.** Abbreviations: *ARv7*: androgen receptor variant seven transcript; cfDNA: cell-free DNA; CNVs: copy number variations; CTC: circulating tumor cell; *ESR1*: gene encoding for the estrogen receptor protein; EV: extracellular vesicle; HER2: receptor tyrosine-protein kinase erb-b2; mRNA: messenger RNA; SNVs: single-nucleotide variants; *SOX17*: SRY-box transcription factor 17.

Analytes			References
CTC	cfDNA	EV	
CTC count, CNVs and SNVs in single CTCs	concentration, tumor fraction, CNVs, SNVs		[126,127]
mutations	mutations		[128]
mutations	mutations		[35]
mutations	mutations		[129]
mutations	mutations		[130]
*ESR1* mutation	*ESR1* mutation		[29]
*ESR1* methylation	*ESR1* methylation		[39]
*SOX17* promotor methylation	*SOX17* promotor methylation		[131]
*SOX17* promotor methylation	*SOX17* promotor methylation		[132]
	5-methylcytosine (5mC) and 5-hydroxymethylcytosine (5hmC)		[133]
mRNA		mRNA	[27]
HER2 protein		HER2 protein	[73]
*ARv7* transcript		*ARv7* transcript	[134]

**Table 2:** **Multimodal liquid biopsy studies.** All publications describing a parallel analysis of more than two liquid biopsy analytes from matched samples are listed and separated according to the clinical purpose. Abbreviations: AR: androgen receptor; BC: breast cancer; CA19-9: carbohydrate antigen 19-9; CEA: carcinoembryonic antigen; cfDNA: cell-free DNA; cfRNA: cell-free RNA; CNVs: copy number variations; CTC: circulating tumor cell; EV: extracellular vesicle; HER2: receptor tyrosine-protein kinase erb-b2; HR: hormone receptor; miRNA: microRNA; mRNA: messenger RNA; MSI: microsatellite instability; NLR: neutrophil-to-lymphocyte ratio; PLR: platelet-to-lymphocyte ratio; RAS: rat sarcoma virus protein/gene; SNVs: single-nucleotide variants; TMB: tumor mutational burden.

Clinical Setting		Analytes				Conclusion	References
Clinical Purpose	Tumor Entity	CTC	cfDNA	EV	Other Analytes		
(1) Technical feasibility							
Technical feasibility	Healthy donors	Count	SNVs and CNVs		Leukocyte count	Protocols established, effects of blood collection tube, blood storage time and sample preparation proven.	[146]
Technical feasibility	Melanoma	Count	Mutations	miRNA	miRNA	Feasible from a single blood collection tube, but the choice of the tube affects the outcome of the analysis.	[147]
(2) Early cancer detection							
Diagnosis	BC (among others)		SNVs and CNVs		Proteins	The combined approach reached a sensitivity of 80% and a specificity of 99% for cancer identification; higher sensitivity compared to single approach.	[143]
Diagnosis	BC (among others)		SNVs, CNVs and Fragmentation			The combined approach detected 91% of patients with cancer; higher sensitivity compared to single approach.	[148]
Diagnosis	BC (among others)		Mutations and Methylation		miRNAs	Combination of cell-free DNA mutations, methylation and miRNAs improved the diagnostic performance of the model.	[149]
Diagnosis	Colorectal		Methylation and CNVs		CEA	Multi-omic approach detected twice as many cancer patients as methylation or CEA analysis alone.	[150]
Diagnosis	Ewing sarcoma and other Pediatric sarcomas		Fragment size, CNVs and Fusion genes			Detection of cfDNA independent of any genetic alterations; this liquid biopsy approach is now more readily accessible for childhood cancers.	[151]
(3) Prognostification							
Initial staging	Pancreas		Concentration, KRAS SNVs		mRNA, miRNA and CA19-9	This combination achieved a sensitivity of 88% and a specificity of 95%; higher sensitivity compared to single approach.	[152]
Prognosis	Pancreas		Concentration, RAS SNVs		NLR, PLR and CA19-9	The combination increased the certainty of the prognostic statement.	[153]
Prognosis	Lung	Count	SNVs	Count		While in only 2% of the patients all these biomarkers were present, at least one of these analytes was detected in 45% of the patients; prognostic value is better with combinational approach.	[145]
Prognosis	Prostate	mRNA and Methylation		mRNA and Methylation		More tumor-associated mRNA profiles in CTCs than EVs. More mRNA expression markers prognostic in CTCs than EVs.	[154]
Prognosis and Therapy guidance	HR + HER2 − MBC	mRNA and SNVs	SNVs	mRNA		Additive value of the analytes for prognosis and therapy decision making.	[155]
(4) Therapy guidance							
Relapse prediction	BC	Count	SNVs, CNVs, TMB, MSI			Highest sensitivity rates to predict minimal residual disease in contrast to the single approaches.	[156]
Molecular characterization	BC	SNVs, Methylation, mRNA				Higher sensitivity of CTC detection by combinational mutation, methylation and mRNA expression profiling compared to CTC identification via protein staining.	[157]
Therapy guidance	Prostate	AR mRNA	AR amp		AR mRNA	Identification of resistance mechanisms in a larger fraction of patients when compared to the evaluation using a single analyte.	[144]
Clinical management	Prostate	Count, SNVs, CNVs	SNVs		cfRNA	CTC and cfDNA analysis reveal distinct data sets; different results of the orthogonal analytes can be explained by the true biological distinctions of the analytes and are highly influenced by methodological factors.	[158]
Therapy guidance and therapy monitoring	Lung under ICI		Concentration (including dynamics) and TMB		T-cells	Each analyte was required for optimal differentiation between responders and non-responders.	[159]
(5) Therapy monitoring							
Therapy monitoring	HR+HER2- MBC	mRNA	SNVs	mRNA		Additive value of the analytes for therapy monitoring and usability of specific analytes for specific clinical purposes.	[160]
Therapy monitoring	MBC	Count, Proteins, SNVs, CNVs	SNVs and CNVs			The tumor evolution of the CNVs can be resolved only within the single CTCs.	[126,127]

## Abbreviations

AR: androgen receptor; BC: breast cancer; CA19-9: carbohydrate antigen 19-9; CEA: carcinoembryonic antigen; cfDNA: cell-free DNA; cfRNA: cell-free RNA; CK: cytokeratin; CNVs: copy number variations; CTC: circulating tumor cell; ctDNA: circulating tumor DNA; ELIMA: Evaluation of multiple liquid biopsy analytes in metastatic breast cancer patients all from one blood sample; EMT: epithelial-to-mesenchymal transition; ERBB2: erb-b2 receptor tyrosine kinase 2; ESR1: estrogen receptor 1; EV: extracellular vesicle; FACS: fluorescence-activated cell sorting; gDNA: genomic DNA; HER2: receptor tyrosine-protein kinase erb-b2; HR: hormone receptor; ICI: immune checkpoint inhibitors; MBC: metastatic breast cancer; miRNA: microRNA; MLR: monocyte-to-lymphocyte; mRNA: messenger RNA; MSI: microsatellite instability; NLR: neutrophil-to-lymphocyte ratio; OS: overall survival; PC: prostate cancer; PD: progressive disease time point; PFS: progression-free survival; PIK3CA: Phosphatidylinositol 4,5-bisphosphate 3-kinase catalytic subunit alpha isoform; PLR: platelet-to-lymphocyte ratio; RAS: rat sarcoma virus protein/gene; snoRNAs: small nucleolar RNAs; SNPs: single-nucleotide polymorphisms; SNVs: single-nucleotide variants; sTK1: serum/circulating thymidine kinase 1; TCR: T-cell receptor; TMB: tumor mutational burden.
